# Flavonoids from *Salvia chloroleuca* with *α*-Amylsae and *α*-Glucosidase Inhibitory Effect

**Published:** 2015

**Authors:** Behvar Asghari, Peyman Salehi, Ali Sonboli, Samad Nejad Ebrahimi

**Affiliations:** a *Department of PlantProduction and Breeding, Faculty of Engineering and Technology, Imam Khomeini International University, Qazvin, Iran. *; b *Department of Phytochemistry, Medicinal Plants and Drugs Research Institute, Shahid Beheshti University, G. C., Tehran, Iran.*; c *Department of Biology, Medicinal Plants and Drugs Research Institute, Shahid Beheshti University, G. C., Tehran, Iran. *; d *Division of Pharmaceutical Biology, University of Basel, Klingelberg Strasse 50, 4056 Basel, Switzerland.*

**Keywords:** *Salvia chloroleuca*, *α*-amylase, *α*-glucosidase, Enzyme inhibition, Flavonoids

## Abstract

It is believed that the inhibition of carbohydrate hydrolyzing enzymes including α-amylase and *α-*glucosidase is one of the therapeutic approaches to decrease the postprandial glucose level after a meal, especially in the people with type 2 diabetes. Medicinal plants and their extracts are one of the main sources to find new inhibitors to the enzymes. In our study four flavonoids, namely luteolin 7-*O*-glucoside (1), luteolin 7-*O*-glucuronide (2), diosmetin 7-*O*-glucuronide (3) and salvigenin (4) were isolated from aerial parts of *Salvia chloroleuca*. The inhibitory activity of these compounds against *α-*amylase and *α*-glucosidase were evaluated. Compounds 1, 2 and 3 showed potent *α*-glucosidase inhibitory effect with IC_50_ values of 18.3, 14.7, and 17.1 µM, respectively. Also these compounds exhibited moderate *α*-amylase activity with IC_50_ values 81.7, 61.5, and 76.3 µM, respectively.

## Introduction

Diabetes mellitus is an emerging health problem worldwide. Recent estimates point to the stark reality that one in three people will have diabetes by 2050 (1). The two major forms of diabetes are type 1 (insulin-dependent) and type 2 (noninsuline-dependent). Type 2 diabetes comprises 90% of people with diabetes around the world, and is largely the result of excess body weight and physical inactivity. It has been demonstrated that in this type of diabetes the blood sugar raises abnormally right after a meal. Therefore, the control of blood postprandial glucose level is an important factor in type 2 diabetes (2). *α*-Amylase and α-glucosidase are the key enzymes catalysing hydrolysis of α-glucosidic bonds in complex carbohydrates, like starch, to liberate absorbable glucose. *α*-Amylase is one of the major products of the pancreas (about 5-6%), that catalyses the initial hydrolysis of starch into shorter oligosaccharides (3). The protein structure of the enzyme contains 3 domains including A, B and C. The catalytic triad (Asp^197^, Glu^233 ^and Asp^300^) is located in domain A. Hydrolysis of starch is thought to proceed via a double displacement reaction with acid-catalyzed formation of a *β*-D-glycopyranosyl-enzyme intermediate followed by base-catalyzed hydrolysis of this linkage. Indeed, Asp^197^ acts as nucleophil that attacks the substrate at the sugar anomeric center and Glu^233^ and Asp^300^ either individually or collectively act as acid or base catalysts (4). *α*-Glucosidase is another enzyme that hydrolyzes terminal non-reducing 1-4 linked α-glucose residues to release a single glucose molecule. It is demonstrated that the residues Trp^516^ and Asp^518^ are critical for catalytic function of the enzyme (5). Inhibition of these two enzymes can retard the release of glucose and effectively control the postprandial elevation of blood glucose level. *α*-Amylase and α-glucosidase inhibitors in current medical use, like acarbose and miglitol, exert serious side effects. Moreover drug resistance is reported for these therapeutic agents (6). To overcome these problems, searching for new α-amylase and *α*-glucosidase inhibitors, especially from natural sources, is underway and recommended (7).

*Salvia* is the largest genus of Lamiaceae which encompasses over 900 species widespread throughout the world, especially in the temperate and warmer zones (8). In Iran, *Salvia* is represented by 58 species, 17 of which are endemic (9). The essential oil and extracts of *Salvia* species possess various biological activities, including, antioxidant, antibacterial, antifungal, antitumor, anti-inflammatory, anticholinesterase, antiprotozoal and antidiabetic activities (8, 10). In Iranian folk medicine some of the *Salvia* species are used for their antidiabetic effect (11). There are also reports about *α*-amylase and α-glucosidase inhibitory effect of *Salvia* species. Various extracts of *S. acetabulosa* were investigated for their α-amylase and α-glucosidase inhibitory activity. Of these, methanol extract with high total phenolic content showed the best inhibition against both enzymes (12). 5-hydroxy-7,4-dimethoxyflavone and oleanolic acid are two effective α-glucosidase inhibitors, isolated from the crude acetone extract of *S. moorcraftiama *(13). Ma *et al*., evaluated the inhibitory effect of *S. miltiorrhiza *Bag. on α-glucosidase and isolated 16 active compounds from the 75% ethanol extract (14). In another study Nickavar and Abolhasani reported the isolation of a flavone, chrysoeriol, from ethanol extract of *Salvia virgata*, which exerted good inhibitory effect against α-amylase (15). The chemical composition and *in-vitro *antimicrobial activity of essential oil of *S. chloroleuca*, an endemic plant of Iran, have been investigated (16). Here we report the inhibitory effect of *S. chlorolueca* extracts on α-amylase and *α*-glucosidase as well as isolation and identification of the active constituents through bioassay guided fractionation procedures. This is the first phytochemical study of *S. chlorolueca* and investigation of its extracts inhibitory abilities against *α*-amylase and *α*-glucosidase.

## Experimental

*Chemicals*

Porcine pancreas α-amylase type VI (EC 3.2.1.1), α-glucosidase type I from Baker’s Yeast (EC 3.2.1.20), 3,5-dinitrosalicylic acid (DNS), *p*-nitrophenyl*-α*-D-glucopyranose (PNPG), maltose and acarbose were obtained from Sigma-Aldrich (Paris, France). Soluble starch, sodium dihydrogenphosphate (NaH_2_PO_4_), sodium potassium tartrate and sodium chloride were purchased from Merck. Analytical grade solvents for extraction and HPLC grade solvents for chromatography were from Scharlau (Barcelona, Spain). HPLC grade water was obtained by an EASY-pure II (Barnstead, Dubuque IA, USA) water purification system. Deuterated solvents were purchased from Armar Chemicals (Döttingen, Switzerland). 

*General*

 Analytical HPLC separations were carried out on a system consisting of a 1100 series binary high-pressure mixing pump with degasser module, column oven and a 1100 series PDA detector (all Agilent, Waldbronn, Germany). A Gilson 215 liquid handler with a Gilson 819 injection module and 50 μL loop was used as autosampler. The HPLC was coupled to an Esquire 3000 Plus ion trap mass spectrometer equipped with an electrospray (ESI) interface (Bruker Daltonics, Bremen, Germany). Data acquisition and processing was performed using HyStar 3.0 software (Bruker Daltonics). Semi-preparative HPLC separations were carried out on an Agilent 1100 series HPLC system consisting of a 1100 series quaternary low-pressure mixing pump with degasser module, column oven, a 1100 series PDA detector, and an autosampler with a 1000 μL loop. The preparative HPLC system consisted of a Shimadzu SCL-10VP controller and binary pump (LC-8A), a UV–vis SPD-M10A VP detector and Class-VP 6.12 as software. NMR spectra were recorded on an Avance III spectrometer operating at 500 MHz and 125 MHz for ^1^H and ^13^C, respectively (Bruker Biospin, Fällanden, Switzerland). A 1 mm TXI probe was used, and data processing was performed with Topspin 2.1 (Bruker). Absorbance of enzyme-assay reaction mixture was measured by BioTek microplate reader (XS2).

*Plant material*

The aerial parts of *S. chloroleuca *Rech. f. & Aell. were collected from Shahrestanak, Tehran province of Iran, in June 2008 at an altitude of 2300 m. The plant was botanically identified by Dr. Ali Sonboli of Biology Department of Medicinal Plants and Drug Research Institute, Shahid Beheshti University, Tehran, Iran. Voucher specimen (MPH 845) has been deposited at the herbarium of Medicinal Plants and Drugs, Research Institute, Shahid Beheshti University, Tehran, Iran.

*Extraction and isolation*

**Figure 1:**
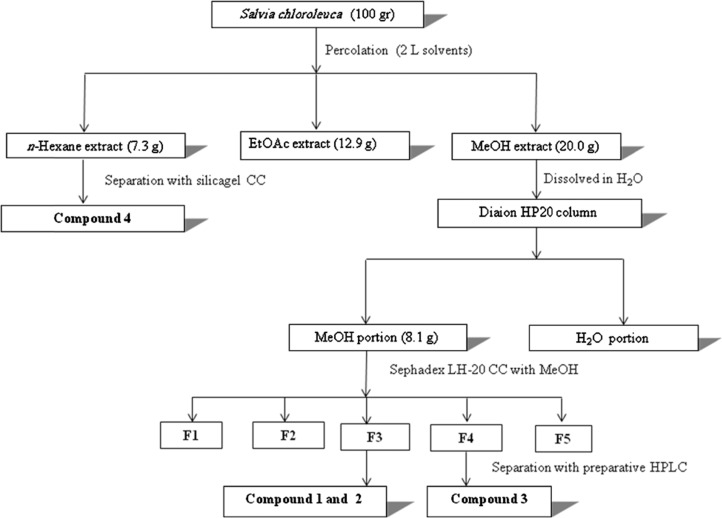
Isolation scheme of *α*-amylase and *α*-glucosidase inhibitors (compounds 1-3) from *Salvia chloroleuca *methanolic extract

Dried leaf material (100 g) was ground with a ZM 1 ultra-centrifugal mill (Retsch, Haan, Germany) equipped with a 0.75 mm Conidur ring sieve, and extracted by successive percolation with *n*-hexane, ethylacetate and methanol (2 L each). After evaporation to dryness under reduced pressure, 20 g of methanol extract was obtained. The extract was suspended in distilled water and loaded onto a Diaion HP-20 column (5 × 40 cm) *i.d*. After washing with water, the column was eluted with methanol (3 L), to provide a fraction enriched in phenolic compounds (8.1 g). This fraction was subjected to column chromatography over sephadex LH-20 (2×50 cm) *i.d*, eluted with methanol. After screening by TLC the obtained fractions with similar compositions were pooled, to yield 5 combined fractions (F1-F5). These main fractions were assayed for their *α*-amylase and α-glucosidase inhibition activities. The most active fractions were separated by preparative HPLC (SunFire C_18_, 5 μm, 150 × 30 mm *i.d*., Waters) with 10-100 % of methanol in water (both containing 0.1 % formic acid), over 40 min at a flow rate of 20 mL/min, and injection volume of 200 µL. Collected peaks from preparative HPLC were evaporated and subjected to semi-preparative HPLC (SunFire C_18_, 5 μm, 150 × 10 mm *i.d*., Waters) with 10-100 % methanol in water (both containing 0.1 % formic acid) over 40 min, at a flow rate of 4 mL/min. Several injections yielded compounds 1 (8 mg), 2 (5 mg) from F3 and 3 (6 mg) from F4. The *n*-hexane extract was separated on silica gel using *n*-hexane-ethylacetate mixtures as eluent. Fractions obtained with 40% ethylacetate (250 mg) were purified by semi-preparative HPLC, and yielded the known compound salvigenin (4) (20 mg). The detailed purification process of active components (1-4) was performed by the flowchart scheme described in Figure 1.

*Luteolin 7-O-glucoside (1)*

^1^H NMR (500 MHz, DMSO-*d*_6_) δ 3.16-3.46 (m, sugar-H), 3.69(d, *J =* 11.0 Hz, H-5″), 5.02 (d, *J =* 7.4 Hz, H-1″), 6.41 (d, *J =* 2.0 Hz, H-6), 6.67 (s, H-3), 6.74 (d, *J =* 2.0 Hz, H-8), 6.87 (d, *J =* 8.3 Hz, H-5′), 7.37-7.40 (m, H-2′,6′). UV λ_max_ 254 nm, 350 nm. MS (*m/z*) 447.1 [M-H]^-^.

*Luteolin 7-O-glucuronide (2)*

^1^H NMR (500 MHz, DMSO-*d*_6_) δ 3.28-3.51 (m, sugar-H), 3.98 (d, *J* = 9.3 Hz, H-5″), 5.23 (d, *J* = 7.2 Hz, H-1″), 6.45 (d, *J* = 2.0 Hz, H-6), 6.70 (s, H-3), 6.79 (d, *J* = 2.0 Hz, H-8), 6.91 (d, *J* = 8.5 Hz, H-5′), 7.40-7.45 (br s, H-2′, 6′). UV λ_max_ 254 nm, 350 nm. MS (*m/z*) 461.1 [M-H]^-^.

*Diosmetin 7-O-glucuronide (3)*

^1^H NMR (500 MHz, DMSO-*d*_6_) δ 3.33-3.45 (m, sugar-H), 3.90 (s, OMe-4′), 4.02 (d, *J* = 9.6 Hz, H-5″), 5.25 (d, *J* = 7.3 Hz, H-1″), 6.47 (d, *J* = 2.0 Hz, H-6), 6.86 (d, *J* = 2.0 Hz, H-8), 6.93 (s, H-3), 6.95 (d, *J* = 8.3 Hz, H-5′), 7.55-7.40 (m, H-2′, 6′). UV λ_max_ 268 nm, 345 nm. MS (*m/z*) 475.1 [M-H]^-^.

*Salvigenin (4)*

^1^H NMR (500 MHz, CDCl_3_) δ 3.89 (s, OMe-4′), 3.92 (s, OMe-7), 3.96 (s, OMe-6), 6.54 (s, H-8), 6.58 (s, H-3), 7.02 (d, *J* = 9.0 Hz, H-3′, 5′), 7.84 (d, *J* = 9.0 Hz, H-2′, 6′). UV λ_max_ 274 nm, 330 nm. MS (*m/z*) 329.1 [M+H]^+^.

*α-Amylase inhibition assay*

*α*-Amylase inhibition activity was assessed by a previously reported procedure with some modifications (17). The assay system, which was carried out in 96-well plates, comprised the following components in a total volume of 250 µL: 100 mM sodium phosphate (pH 6.8), 17 mM NaCl, 1.5 mg soluble starch, 50 µL of inhibitor solution in DMSO at various concentrations (for pure compounds 12.5, 25, 50, 100 and 150 µM), and 10 µL of enzyme solution (25 unit/mL). After incubation at 37 °C for 30 min, the reaction was stopped by addition of 20 µL NaOH (2N) and 20 µL color reagent (4.4 µM of 3,5-dinitrosalisylic acid, 106 µM of potassium sodium tartarate tetrahydrate and 40 µM of NaOH) followed by a 20 min incubation at 100 °C water bath. *α*-Amylase activity was determined by measuring the absorbance of the mixture, due to the maltose generated at 540 nm. Individual blanks were prepared to correct for the blank ground absorbance, where the enzyme was replaced with buffer as follows:

Corrected absorbance of test sample = Absorbance of sample –absorbance of blank

From the net absorbance obtained, the % (w/v) of maltose generated was calculated from the equation obtained from the maltose standard calibration curve (0–0.1%, w/v, maltose).

Control incubations, representing 100% enzyme activity, were conducted in the same manner replacing the plant extract with DMSO. The percentage of α-amylase inhibition was calculated by the following equations:

$\mathrm{\% reaction}=\frac{\mathrm{mean of maltose in sample}}{\mathrm{mean of malt}\mathrm{ose in control}}\times 100$

% α-amylase inhibition activity= 100 - % reaction

*α-Glucosidase inhibition assay*

The α-glucosidase inhibition was measured according to an earlier reported bioassay method (18). The mixture contained 20 µL* α*-glucosidase (0.5 unit/mL), 120 µL of 0.1 M phosphate buffer (pH 6.9) and 10 µL of test sample at varying concentrations (for pure compounds 5, 10, 15, 30 and 50 µM). The mixed solution was incubated in 96-well plates at 37 °C for 15 min. After preincubation, the enzymatic reaction was initiated by adding 20 µL of 5 mM *p*-nitrophenyl-*α*-D-glucopyranoside solution in 0.1 M phosphate buffer (pH 6.9), and the reaction mixture was incubated for another 15 min at 37 °C. The reaction was stopped by adding 80 µL of 0.2 M sodium carbonate solution and then the absorbance was measured by microplate reader at 405 nm. The reaction system without plant extracts was used as control and the system without *α*-glucosidase was used as blank for correcting the background absorbance. The inhibitory rate of sample on* α*-glucosidase was calculated by the following formula:

$\mathrm{\% Inhibition}=\left(\frac{\mathrm{control absorbance}-\mathrm{sample absorbance}}{\mathrm{control absorbance}}\right)\times 100$

***Statistical Analysis***

Statistical analyses were done using GraphPad Prism version 5.00 for Windows. Differences were evaluated by one-way analysis of variance (ANOVA) test completed by Tukey’s multicomparison test. Statistical signiﬁcance was declared at a *p*<0.05. All assays were performed at least in triplicate and the results were expressed as mean ± standard deviation (SD). IC_50_ values were determined by plotting a percent inhibition versus concentration curve for all assays.

## Results and Discussion

The α-amylase and *α*-glucosidase inhibitory effect of *n*-hexane, ethyl acetate and methanol extracts of *S. chlorolueca* were evaluated. The results are shown in Table 1. The methanolic extract possessed strong inhibitory activities against both α-amylase and α-glucosidase with IC_50_ values of 39.8 and 13.3 µg/mL, respectively. Also *n*-hexane extract showed moderate *α*-glucosidase activity with IC_50_ of 26.2 µg/mL. The high activities of the crude extracts prompted us to find the active compounds.

**Figure 2:**
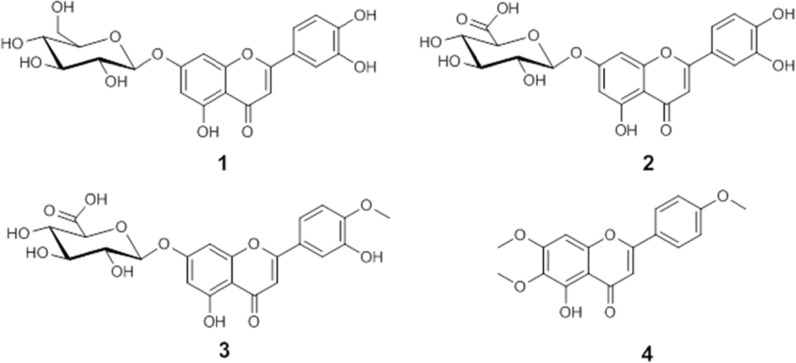
Chemical structures of isolated compounds.

Bio-assay guided fractionation of active extracts led to the isolation of luteolin 7-*O*-glucoside (1), luteolin 7-*O*-glucuronide (2), and diosmetin 7-*O*-glucuronide (3) as active compounds of methanolic extract, whereas salvigenin (4) was the major compound isolated from *n*-hexane extract. Isolated compounds were identified by ESI-MS, 1D and 2D NMR spectroscopy, and by comparison with published data (19-22). Chemical structures of the compounds are shown in Figure 2.

**Table 1:** *α*-amylase and α-glucosidase inhibition activities of *S. chlorolueca *extracts.

Extract of *S. chlorolueca*	*α*-amylase (IC_50_ µg/mL)	*α*-glucosidase (IC_50_ µg/mL)
*n*-hexane	57.0 ± 1.2	26.2 ± 3.2
ethyl acetate	62.2 ± 3.4	37.9 ± 2.7
methanol	39.8 ± 1.9	13.3 ± 1.3

The isolated compounds were further examined for the inhibition of *α*-amylase and α-glucosidase. Table 2 shows the inhibitory activity of the isolates. Compounds 1, 2 and 3 had a moderate inhibition effect on *α*-amylase with IC_50_ value of 81.7 µM (1), 61.5 µM (2), and 76.3 µM (3), respectively, while the IC_50_ value for acarbose against *α*-amylase was 53.4 µM. Among the isolates, compound 2 showed excellent inhibition on *α*-glucosidase with extremely high potency and its IC_50_ value was 14.7 µM, while the IC_50_ value for acarbose was 16.1 µM. Compounds 1 and 3, also exhibited potent inhibition against *α*-glucosidase with IC_50_ values of 18.3 and 17.1 µM, respectively. Salvigenin (4) as the major compound of *n*-hexane extract exhibited a poor inhibitory activity on both enzymes. Its IC_50_ values were calculated more than 100 and 50 µM for *α*-amylase and *α*-glucosidase, respectively. 

The comparison of the isolated compounds inhibitory effect on the enzymes showed that the hydroxyl substitution on the B ring as well as presence of sugar moiety on A ring are effective factors in the inhibitory activity of the flavonoids. As it is shown in Table 2, the absence of these factors in compound 4 dramatically reduced the inhibition effect against α-amylase and *α*-glucosidase. 

**Table 2:** Inhibition of *α*-amylase and *α*-glucosidase by isolated compounds

**Compounds**	***α*** **-amylase (IC**_50_ ** µM)**	***α*** **-glucosidase (IC**_50 _ **µM)**
(**1**)	81.7 ± 2.3*	18.3 ± 1.7
(**2**)	61.5 ± 1.4	14.7 ± 2.1
(**3**)	76.3 ± 0.6*	17.1 ± 1.9
(**4**)	>100	>50
Acarbose	53.4 ± 3.1	16.1 ± 0.8

* Each value represents the mean±SD, statistically significance compared with acarbose (*p*<0.05 as compared with acarbose).

It was reported that luteolin 7-*O*-glucoside (1) is a strong α-amylase and *α*-glucosidase inhibitor, and our results confirm this finding (23). Changing of the glucose moiety to glucuronic resulted in increasing inhibitory activity on *α*-amylase and α-glucosidase (1 and 2). It is suggested that the interaction between flavonoids and enzymes occurs with the fraction of hydrogen bonds via the sugar or hydroxyl groups in positions C-6 or C-7 of the ring A and position C-4׳ and C-5׳ of the ring B, with the active site (24). It can be concluded that the ability of carboxylic acid group (2) in the formation of hydrogen bond with the active site of the enzymes, is better than that of hydroxyl group (1). The lower ability of compound 3 (diosmetin 7-*O*-glucuronide) in inhibition of α-amylase and *α*-glucosidase than that of compound 2 (luteolin 7-*O*-glucuronide) can be related to the methoxyl substitution of C-4׳. Tadera *et al.* (2006), also reported that the presence of methoxyl group on C-4׳ was unfavorable to the inhibitory activity of flavonoids against α-amylase and *α*-glucosidase, showing that 4׳-OH was important to the inhibitory effect (24).

The main drawback of the currently used drugs, like acarbose for inhibition of digestive key enzymes, is their side effects such as bloating, meteorism and flatulence caused by abnormal bacterial fermentation of undigested carbohydrates in the colon (25). It has been suggested that strong inhibitors of *α*-glucosidase with mild inhibitory activity against α-amylase can overcome this challenge (2). Compounds 1, 2 and 3 showed high inhibitory activity on *α*-glucosidase and moderate inhibitory activity on *α*-amylase, suggesting that they could be employed as a model for the design of new drugs for treatment of diabetes with minimal side effects. Further experiments should be carried out on *in-vivo* tests and on the mechanism and kinetic studies of individual flavonoids. 

## References

[1] Boyle JP, Thompson TJ, Gregg EW, Barker LE, Williamson DF (2010). Projection of the year 2050 burden of diabetes in the U.S. adult population: dynamic modelling of incidence, mortality, and pre-diabetes prevalence. Popul. Health Metr.

[2] Dong HQ, Li M, Zhu F, Liu FL, Huang JB (2012). Inhibitory potential of trilobatin from Lithocarpus polystachyus Rehd against α-glucosidase and α-amylase linked to type 2 diabetes. Food Chem.

[3] Whitcomb DC, Lowe ME (2007). Human pancreatic digestive enzymes. Digest. Dis. Sci.

[4] de Sales PM, de Souza PM, Simeoni LA, de Oliveira Magalhães P, Silveira D (2012). α-Amylase inhibitors: a review of raw material and isolated compounds from plant source. J. Pharm. Pharm. Sci.

[5] Hermans MM, Kroos MA, van Beeumen J, Oostra BA, Reuser AJ (1991). Human lysosomal α-glucosidase characterization of the catalytic site. J. Biol. Chem.

[6] Ghosh S, Ahire M, Patil S, Jabgunde A, Dusane MB, Joshi BN, Pardesi K, Jachak S, Dhavale DD, Chopade BA (2012). Antidiabetic activity of Gnidia glauca and Dioscorea bulbifera: potent amylase and glucosidase inhibitors. Evid. Based Complement. Alternat. Med.

[7] World Health Organization (2002). WHO Traditional Medicine Strategy.

[8] Rustaiyan A, Masoudi S, Tabatabaei Anaraki M (2007). Terpenoids from Iranian Salvia species. Nat. Prod. Commun.

[9] Mozaffarian V (1996). A dictionary of Iranian plant names.

[10] Moridi Farimani M, Bahadori MB, Taheri S, Ebrahimi SN, Zimmermann S, Brun R, Amin G, Hamburger M (2011). Triterpenoids with rare carbon skeletons from Salvia hydrangea: antiprotozoal activity and absolute configurations. J. Nat. Prod.

[11] Zargari A (1997). Medicinal Plant.

[12] Loizzo MR, Saab AM, Tundis R, Menichini F, Bonesi M, Piccolo V, Statti GA, Cindio B, Houghton PJ, Menichini F (2008). In-vitro inhibitory activities of plants used in Lebanon traditional medicine against angiotensin converting enzyme (ACE) and digestive enzymes related to diabetes. J. Ethnopharmacol.

[13] Khan T, Zahid M, Asim M, Hassan S, Iqbal Z, Iqbal Choudhary M, Uddin Ahmad V (2002). Pharmacological activities of crude acetone extract and purified constituents of Salvia moorcraftiana Wall. Phytomed.

[14] Ma HY, Gao HY, Sun L, Huang J, Xu XM, Wu LJ (2011). Constituents with α-glucosidase and advanced glycation end-product formation inhibitory activities from Salvia miltiorrhiza Bge. J. Nat. Med.

[15] Nickavar B, Abolhasani L (2013). Bioactivity-guided separation of an α-amylase inhibitor flavonoid from Salvia virgata. Iran. J. Pharm. Res.

[16] Yousefzadi M, Sonboli A, Ebrahimi SN, Hashemi SH (2008). Antimicrobial activity of essential oil and major constituents of Salvia chloroleuca. Z. Naturforsch.

[17] Miller GL (1959). Use of dinitrosalicylic acid reagent for determination of reducing sugar. Anal. Chem.

[18] Zhang L, Hogan S, Li J, Sun S, Canning C, Zheng SJ, Zhou K (2011). Grape skin extract inhibits mammalian intestinal α-glucosidase activity and suppresses postprandial glycemic response in streptozocin-treated mice. Food Chem.

[19] Lu Y, Foo LY (2000). Flavonoid and phenolic glycosides from Salvia offcinalis. Phytochem.

[20] Murata T, Miyase T, Yoshizaki F (2010). Cyclic Spermidine alkaloids and flavone glycosides from Meehania fargesii. Chem. Pharm. Bull.

[21] Gohari AR, Ebrahimi H, Saeidnia S, Foruzani M, Ebrahimi P, Ajani Y (2011). Flavones and flavone glycosides from Salvia macrosiphon Boiss. Iran. J. Pharm. Res.

[22] Ayatollahi SA, Shojaii A, Kobarfard F, Mohammadzadeh M, Choudhary MI (2009). Two flavones from Salvia leriaefolia. Iran. J. Pharm. Res.

[23] Kim JS, Kwon CS, Son KH (2000). Inhibition of alpha-glucosidase and amylase by luteolin, a flavovonoid. Biosci. Biotechnol. Biochem.

[24] Tadera K, Minami Y, Takamatsu K, Matsuoka T (2006). Inhibition of α-glucosidase and α-amylase by flavonoids. J. Nutr. Sci. Vitaminol.

[25] Bischoff H (1994). Pharmacology of alpha-glucosidase inhibition. Eur. J. Clin. Invest.

